# Deconstructing anti-harm-reduction metaphors; mortality risk from falls and other traumatic injuries compared to smokeless tobacco use

**DOI:** 10.1186/1477-7517-3-15

**Published:** 2006-04-18

**Authors:** Carl V Phillips, Brian Guenzel, Paul Bergen

**Affiliations:** 1 University of Alberta, Department of Public Health Sciences. 215 College Plaza, Edmonton, Alberta T6G 2L9, Canada; 2 Center for Philosophy, Health, and Policy Sciences 10923 Atwell Dr, Houston TX 77096, USA; 3 University of Alberta, Department of Public Health Sciences. 13-103 Clinical Sciences Building, Edmonton, Alberta T6G 2G3, Canada

## Abstract

Anti-harm-reduction advocates sometimes resort to pseudo-analogies to ridicule harm reduction. Those opposed to the use of smokeless tobacco as an alternative to smoking sometimes suggest that the substitution would be like jumping from a 3 story building rather than 10 story, or like shooting yourself in the foot rather than the head. These metaphors are grossly inappropriate for several reasons, notably including the fact that they are misleading about the actual risk levels. Based on the available literature on mortality from falls, we estimate that smoking presents a mortality risk similar to a fall of about 4 stories, while mortality risk from smokeless tobacco is no worse than that from an almost certainly non-fatal fall from less than 2 stories. Other metaphors are similarly misleading. These metaphors, like other false and misleading anti-harm-reduction statements are inherently unethical attempts to prevent people from learning accurate health information. Moreover, they implicitly provide bad advice about health behavior priorities and are intended to persuade people to stick with a behavior that is more dangerous than an available alternative. Finally, the metaphors exhibit a flippant tone that seems inappropriate for a serious discussion of health science.

## Background

Harm reduction refers to health-improving strategies that attempt to replace a highly unhealthy exposure (behavior, etc.) with one that is considerably less unhealthy, though generally not harmless (thus "harm reduction" rather than "harm elimination"). For example, smokers can eliminate almost all of the disease risk from their tobacco use by switching from cigarettes to smokeless tobacco (e.g., snuff dipping), without facing the prospect of giving up nicotine.

Anti-harm-reduction activists sometimes offer pseudo-analogies to try to ridicule the very idea of harm reduction strategies. Two of these inappropriate metaphors used most often in the context of smokeless tobacco (ST) are that switching from smoking cigarettes to ST is like jumping from the 3rd floor of a building rather than the 10th (the actual numbers vary), or shooting yourself in the foot instead of shooting yourself in than the head. Both of these exhibit a flippant tone that seems inappropriate for a serious discussion of health science (despite which, the dean of a U.S. school of public health actually used one of these as part of his rationalization when preventing one of us from pursuing harm reduction research as a faculty member there). Moreover, the analogies are factually flawed and thus fundamentally misleading. The main purpose of this paper is to document the latter point.

## Methods, results, and discussion

### Mortality associated with uncontrolled falls

Probably the most viewed anti-harm-reduction metaphor was on the U.S. television program, *Good Morning America*, in 1994, when in response to Brad Rodu and his then-novel message about ST-based harm reduction, Gregory Connolly, director of the Massachusetts [USA] Tobacco Control Program compared it to jumping from the 3rd floor rather than the 10th [1]. More recent versions of the metaphor were uncovered by our systematic review of information about the risks of smokeless tobacco on the world wide web (described in detail in [2]) and subsequent *ad hoc *internet searchers for key phrases in online news stories and web pages. The several versions we found varied substantially, but likened smoking to falls from at least the 10th floor and ST to falls from at least the 3rd; we found numbers as high as 50 and 30.

While the danger from a fall from a given height varies substantially with the victim's age, physical abilities, landing attitude, and, especially, the surface landed upon, anyone with passing familiarity with the human body and Earth's gravity should be aware that falls from the 10th story (about 35 meters, calculating a bit under 4 meters per story in tall buildings, subtracting a bit for the window being lower than the top of the story) are almost always fatal. Thus, every version of the metaphor we have seen is absurd, with the greater distance fallen considerably worse for someone's health than smoking (or any other behavior imaginable). More importantly, the lower number grossly exaggerates the risk from ST.

Estimates of the portion of smokers whose death is substantially hastened by their smoking range as high as 1/2 in wealthy countries, down to 1/3 or 1/4. Sorting out claims of upward bias in these estimates and correcting for deaths that were not significantly hastened compared to competing causes is difficult, but these considerations argue for the lower end of this spectrum. For smokeless tobacco, even the worst-case-scenario estimates are no higher than 1/10th of that of smoking [3], though there is no basis in the epidemiologic evidence to suggest it is nearly that high. There is better support for the claim that it is in the range of 1/100th, perhaps 2/100ths [4], perhaps less [5]. We will use the rough approximations (adequate for present purposes) of a substantially premature death rate of 1-in-4 from smoking (which might understate the contrast between smoking and ST, since most estimates are higher than this) and 1-in-400 from ST. The latter figure assumes that ST causes *some *mortality, despite the lack of epidemiologic evidence compellingly linking it to any specific life-threatening disease. (The limits of our science make it impossible to distinguish between risks that add to 1-in-400 from, say, 1-in-50 or 1-in-one million; fortunately, the central theses of this analysis do not depend on the exact magnitude.)

To estimate the height of falls that cause similar mortality rates, we conducted a review of the available literature on mortality rates as a function of free fall distance (we did not consider fatal slips that do not involve free fall, such as head injuries from falls in the bath or hip fractures in the elderly when falling from standing). It is surprising how little information is published on the topic. As with most of the health literature, dissimilar exposures are usually lumped together and more attention is given to the exceptional cases and outliers (e.g., very long falls that were miraculously survived) than to representative information. But we were able to identify a few useful sources of information [6-9]. The literature suggests that falls from up to the 3rd story (the rather fuzzy unit of building stories is usually used to measure height) are most always survived, with the death rate increasing sharply and approaching 100% over the next three or four stories.

Our best (admittedly somewhat rough) estimate from the available literature is that a 1-in-4 mortality rate is reached in the range of the 5th story window, while a 1-in-400 mortality rate is reached somewhere short of a 3rd story window. The first author's experience with rock climbing, and the lore thereof, tends to support the latter estimate: falls of ~9 meters onto flat ground are seldom fatal, but occasionally they are. Thus, the largest numbers that could be justified for use in the metaphor are roughly 5th and 3rd story windows for the two products.

Notice an immediate implication of this is that the 10 and 3 story comparison dramatically overstates the absolute risk reduction (risk difference) from switching from cigarettes to ST. Assuming that suicide is not actually one's goal (and tobacco users are not generally trying to kill themselves despite rhetoric to the contrary – a fact that seems to be conceded by users of the metaphor), choosing to jump from the 3rd story rather than the 10th is a very good choice indeed.

Some advocates who use higher numbers of stories are intentionally making the absurd claim that tobacco use (in any form) is virtually always fatal. By contrast, users of the metaphor who use less absurd heights like the 3rd and 10th story are typically conceding that there is a difference in risk (perhaps not realizing its magnitude), but insist that the less risky exposure is still so bad that it should not be proposed as an alternative. If we ignore the dubious premise that a major reduction in harm is not worthwhile because the residual risk is still high (consider, e.g., seatbelts), this point hinges on the absolute risk from ST, not the relative risk. For this, the 3rd story comparison is still misleading.

The above comparison of a lifetime of ST use to a 3rd story fall was based on the probability of the exposure being a contributing factor in a premature death, whenever the death might occur. A better measure of the cost of an exposure is years of potential life lost. This is important because mortality that results from a fall (or gunshot) almost always occurs almost immediately, while deaths from chronic exposures tend to occur far in the future, at an age when death from a competing cause will occur sooner. Even without correction for time preference (discounting), we estimate that for individuals in the target audience, deaths from falls cost in the order of five times as many life years as deaths from contemporarily taking up (or continuing) a lifetime course of smoking. Correcting for this, the appropriate falling distances are closer to the 4th story window or a bit lower for smoking and in the range of 2nd story for ST.

These more accurate analogies might actually be fairly useful in painting the picture for consumers. A nontrivial portion of young men (particularly the "risk takers" who are more inclined to use tobacco [10]) have probably jumped from a 2nd story window, but few would dare jump from the 4th. To keep the metaphor catchy and less burdened with numbers, we might suggest that the harm-reduction decision is like foregoing a jump from the top of the roof of a large house, opting to instead jump from the garage. It seems like an easy choice, as well as a useful metaphor since, again, many people have jumped from the roof of a small garage, but few could bring themselves to jump from the peak of the roof of a three story house.

It should be noted that nonfatal falls from heights often cause morbidity, but we make no attempt to incorporate this into our calculation of comparative mortality. There is a fair probability of injury from a non-fatal 2-story fall, making the prospect of such a fall intimidating, and so the analogy remains an overstatement of the morbidity risk when mortality risk is equalized. Thus, even the 2nd and 4th stories tends to overstate the risk from ST and possibly from smoking (though the latter is known to cause various non-fatal major morbidities at a high rate, so that contrast is not so large).

### Mortality from self-inflicted gunshot wounds

It is immediately obvious that the gunshot metaphor is absurd for the same reasons as the 10-vs-3 version of the jumping metaphor: If someone was faced with the choice of shooting himself in the head and ..., well, almost anything else, really, but in particular shooting himself in the foot or leg, the latter option is quite obviously better from a health outcomes perspective. Again, the attempt to ridicule harm-reduction actually makes a pretty good case for it.

Beyond that, however, the analogy fails. Mortality risk from self-inflicted gunshot wounds to the head dwarfs that from smoking, while foot wounds, though they have a low mortality rate, have a high probability of permanent debilitating orthopedic damage, a risk absent in tobacco use. A penetrating gunshot wound to the upper leg stands a nontrivial chance of being fatal, greater than the risk from ST.

We hesitate to try to provide a correction, like the above corrected height of fall, concerning where in one's body to inflict a gunshot wound to cause a certain probability of mortality. Self-inflicted gunshot wounds unambiguously evokes attempted suicide. The psychological health problems that lead to such behaviors make it seem inappropriate to either use this as a whimsical attack on harm reduction, or to try to deconstruct the joke in any further detail. (Though it is interesting to note that self-administration of nicotine is thought to provide relief from certain psychological and neurodegenerative diseases that could lead to self-inflicted trauma if untreated [11,12].) Moreover, blithe references to shooting oneself seem particularly inappropriate with hundreds of thousands of members of the main target population (most of the rhetoric originates in the USA and is directed at adolescents) are experiencing the horrors of pointless military combat and its various deadly tools, with quite a few committing suicide following the experience [13-15]. (As an aside: it is likely that tens of thousands of young men and women took up smoking during their recent combat experience, increasing the importance of harm reduction.)

### Other metaphors

Our research uncovered a handful of less common metaphors, which have either fallen from favor or never caught on. Getting hit by a car rather than a truck (found in a few dental health websites, attributed to a single source [16]) is another case where there is no appreciable difference in the risks from the exposures *ceteris paribus*, though the details of the collision are so obviously important that the metaphor is hopelessly muddled. Shooting oneself with one type of military weapon rather than another (e.g., a rifle rather than an Uzi [17]) also seems to have not caught on. This one likely failed to gain popularity because the location of the wound is paramount, and the level of detail makes it enough more distasteful so that even those who like the generic gunshot metaphor might avoid it.

One of the more bizarre metaphors we found in our research is that switching to ST is like driving at 100 miles per hour rather than 140 miles per hour [18]. The mortality rate from taking a car up to one of these speeds is quite trivial; people do it rather frequently. Perhaps the authors meant driving that speed all the time, but this seems prohibitively challenging. The reason this one is worth mentioning is because it reminds us that people who use tobacco are well known to be more likely to have a variety of risky and unhealthy behaviors, such as habitually driving dangerously. Every time health advocates expend effort and use up people's limited attentiveness to health matters trying to dissuade them from using ST, it not only reduces the prospect of harm reduction, but reduces the potential to affect other behaviors that are much riskier.

## Conclusion

Rather than actually debating the costs and benefits of proposals for harm reduction policies, opponents often display a non-scientific zeal which leads them to forgo substantive argument and use whatever rhetoric they think might shut down the discussion. If there are substantive arguments to be made against a harm reduction proposal, they should certainly be introduced into open debate. But exaggerated metaphors do not qualify as substantive arguments and violate the ethical duty (incumbent on all who claim some mantle of expertise and provide health advice) to provide people with accurate health information rather than trying to mislead or manipulate them.

The manipulation is especially relevant in light of an additional problem with all these metaphors: By comparing tobacco use to acts that are basically only about hurting oneself, anti-harm-reduction advocates (and anti-tobacco advocates more generally) try to imply that the most salient feature – or even the only important feature – of tobacco use is the mortality risk. People use tobacco and engage in other behaviors because they have decided that a certain consumption pattern gives them greater net benefits than any alternative they have considered. The metaphors, like much of health advocacy, implicitly claim that people ought to be concerned about mortality risk to the exclusion of all else, and try to define the terms of the discussion to reinforce that premise. (We recognize that we risk falling into their trap by even challenging the details of the metaphors.) Certain health advocates believe it is acceptable to mislead people into making choices they would not otherwise make; presumably they rationalize this based on the absurd premise that delaying mortality is the only thing that matters. Trying to change people's behavior through risk communication is an ethical and very legitimate health promotion activity, assuming it is based on giving people accurate information for making their own choices. It is fundamentally unethical when it consists of making the choices for them and trying to trick them into complying.

Through the use of various tactics, advocates who oppose the use of ST as a harm reduction tool have managed to convince most people that the health risk from ST is several orders of magnitude greater than it really is [19,20]. The primary tactic they use is making false or misleading scientific claims that suggest that all tobacco use is the same [2]. But those who wish to provide consumers with honest information and options should not ignore other tactics used by anti-harm-reduction advocates. Metaphors are a potentially effective tactic for misleading the public. They can serve as a politician-style soundbite, dominating typical fluff news stories and sticking in people's memory.

A potentially effective – and certainly appropriate – response to health-damaging metaphors is to respond as if they were serious scientific claims and analyze them accordingly. It is difficult to see how the purveyors of the metaphors could respond without looking absurd. An honest response on their part – that it is meant to be a catchy little joke, or that accuracy does not matter when trying to persuade people – should ring rather hollow, given the deadly seriousness of what health advocates who make unjustified attacks on harm reduction are doing: Apparently motivated by their hatred of all things tobacco, they are trying to convince people to not switch from an extremely unhealthy behavior to an alternative behavior that eliminates almost all of their risk. Who is shooting whom in the foot?

## Abbreviations

ST = smokeless tobacco

## Competing interests

The author(s) are interested in promoting the use of ST as a harm reduction strategy for improving the public health and in promoting more ethical health communication. Both of these goals should be evident from the text. CVP is also actively engaged in promoting better quantitative health policy analysis. We have no interests that we consider to compete with these goals. This research was supported by an unrestricted gift from U.S. Smokeless Tobacco Company (currently to the University of Alberta, formerly to the Center for Philosophy, Health, and Policy Sciences, Inc.) for support of the research of Dr. Phillips and colleagues. The funder exercises no influence over our choice of research or presentation of findings, and will not be aware of this paper until it is published. CVP has also worked as a consultant for USSTC in connection with litigation.

## Authors' contributions

CVP was the primary author of the text, conducted some of the research, and oversaw the remainder of the research. BG conceived of the idea for this paper and conducted early research. PB conducted later research and contributed to the drafting of the text.
